# How to Remove an Eyeball and the Treatment for Excessive Hemorrhage from the Operation—Also a Report of a Case of Hemorrhage from Clipping the Tonsil

**Published:** 1890-02

**Authors:** J. M. Crawford

**Affiliations:** Assistant to Chair of Diseases of Eye, Ear, Throat and Nose, Atlanta Medical College, Atlanta, Ga.


### Clinical Dpartment

### HOW TO REMOVE AN EYEBALL AND THE TREATMENT FOR EXCESSIVE HEMORRHAGE FROM THE OPERATION—ALSO A REPORT OF A CASE OF HEMORRHAGE FROM CLIPPING THE TONSIL.

J. M. CRAWFORD, M. D., ASSISTANT TO CHAIR OF DISEASES OF EYE, EAR, THROAT AND NOSE, ATLANTA MEDICAL COLLEGE, ATLANTA, GA.

The operation of removing an eye-ball is not an easy one when the operator has no fixed idea about what steps are to be made and when to make them. You will pardon me, therefore, for telling how I like to make the operation.

Having etherized the patient and dropped in a few drops of a 10 per cent. solution of muriate cocaine on the ball, you place yourself in front of him. If it be his left eye to be enucleated, sit on his left side—if his right eye, sit on the patient's right. This being done, arrange your eye-speculum and with a pair of forceps grasp the conjunctiva and with a pair of curved scissors clip it around the corneo-sclerotic margin—then grasp the external rectus if it is his left eye, but the internal rectus if it is his right eye and clip it—taking care not to let the forceps loose—keeping the ball steady by the grasp you already have, you cut the superior rectus and then the inferior rectus; you now with your scissors feel for the optic nerve, which you will readily find and clip it near the ball. Now with your scissors dislocate the ball, lay your forceps down, as you can manage it better with your fingers—cut the remaining muscles as you see them.

When these steps are taken the operation is a very simple one.

As a rule, there is but little danger to be feared from hemorrhage from the operation unless there is a considerable abnormal situation of the opthalmic artery or greatly increased size of the arteria centralis retinæ.

Should hemorrhage occur, however, firm pressure with a few sponges over the closed eye-lid will *always* stop it. You have here hemorrhage occurring in a bony cavity and pressure is bound to relieve it.

The objection to the application of the muriated tinc. iron is that it is not so easily done, and it stains everything with which it comes in contact.

On January 17th, E. D., aged 14—white; presented himself with chronic enlarged tonsils. On examination, it was found that the right tonsil was much the larger and we decided to remove only that one. The operation was made with a volcella forceps and curved bistory—cutting from below upwards.

No undue amount of hemorrhage occurred immediately after the operation; but in a short time it increased and continued for six hours, with the exception of the time when pressure was applied to the cut surface.

Various means were tried to stop it, such as a gargle of turpentine, salt water, and the application of Tr. Chlor. Iron, mopping throat with a strong solution of Tannic Acid, also torsion; but none was found so effectual as pressure on the cut surface with a wet sponge. As long as this was done there was no bleeding, and it entirely ceased after a pressure of two hours and a half.

The young man went home, and had no more hemorrhage for four days, at which time he removed a large clot from his throat, and the bleeding commenced and continued for several hours until he fainted. There has been no hemorrhage now for four days, and his father, who is a physician, writes me he is much stronger and that the throat looks much better. I feel satisfied that he will have no more trouble, and that he will soon be himself again. This is the first case of the kind I have seen, and there are but few on record.

I am at a loss to know the cause of the hemorrhage, unless it was from a patulous condition of the arteries from the tonsil being very hard and tough—or an abnormal position of the artery.

